# Practice and determinants of emergency contraceptive utilization among women seeking termination of pregnancy in Northwest Ethiopia—A mixed quantitative and qualitative study

**DOI:** 10.1371/journal.pone.0263776

**Published:** 2022-02-11

**Authors:** Lebeza Alemu Tenaw

**Affiliations:** Department of Reproductive Health, College of Health Sciences, Woldia University, Woldia, Ethiopia; Illawarra Shoalhaven Local Health District, AUSTRALIA

## Abstract

**Background:**

Emergency contraceptives are used within 72 hours after unprotected sexual intercourse to prevent unwanted pregnancy. Although emergency contraceptives are widely available in Ethiopia, termination of pregnancy remains a public health problem indicating low uptake of emergency contraceptives after unprotected sexual intercourse. This study aimed to assess utilization and determinants of emergency contraceptives among women seeking termination of pregnancy in Northwest Ethiopia.

**Methods:**

An institutional-based cross-sectional study was carried out, supplemented by phenomenologically approached in-depth interviews. Systematic random sampling was used to select study participants. A structured questionnaire and an in-depth interview guide were used to collect data. Data were entered by EPI-info and analyzed through SPSS version 23 to conduct logistic regressions. Thematic analysis was used to conduct qualitative interpretation.

**Results:**

Almost one-fifth (78; 19.2%) of the study participants used emergency contraceptives to prevent their index pregnancy. Women who had secondary education (aOR 3.28; 95% CI 1.59, 6.79) and women who had no living children (aOR 4.52; 95% CI 1.40, 14.57) had a positive significant association with emergency contraceptive utilization. On the other hand, women who did not discuss contraceptives with their sexual partner (aOR 0.49; 95% CI 0.27–0.91) and women without a history of abortion (aOR 0.45; 95% CI 0.24–0.97) had a negative significant association with emergency contraceptive utilization.

**Conclusion and recommendation:**

There is relatively low utilization of emergency contraception among pregnancy terminating women. Reproductive health programs should encourage women discussion with their partners about emergency contraceptives to decrease occurrence of unwanted pregnancy and termination of pregnancy.

## Background

Unwanted pregnancy is preventable up to 99% by using emergency contraceptives within 72 hours after unprotected sexual intercourse [1, 2]. Emergency contraceptives are used after failure of barrier contraceptive methods, sexual assault, and missed oral contraceptive pills [3]. Effective contraception has profound benefits by decreasing maternal mortality and morbidity, empowering women to reach their own choices about fertility, and increasing women’s economic status [4].

Worldwide young women and men suffer a disproportional share of unplanned pregnancies, termination of pregnancy, and other serious reproductive health problems. In low-income countries, 44% of all pregnancies in 2014 were unintended, 55% of which ended in abortion [5]. In Ethiopia, around 620,300 induced abortions were performed in 2014. The rate of induced abortion in health institutions was increased persistently from 27% to 53% between 2008 and 2014 among all pregnancies [6]. Treatment received for complications of abortion in Ethiopia nearly doubled between 2008 and 2014, rising from 52,600 to 103,600 (8).

To alleviate complications from unwanted pregnancy, emergency contraceptives can have a benefit [7]. Africa needs a comprehensive contraceptive program to prevent unwanted pregnancies and maternal mortality associated with termination of pregnancy [8]. Three quarters of unplanned pregnancies can be prevented by using emergency contraceptives [9].

Although emergency contraceptives were introduced in Ethiopia before two decades, recent studies showed very low utilization of emergency contraceptives after unprotected sexual intercourse [10–12]. Despite widespread availability of family planning service, induced termination of pregnancy remains a public health problem due to poor contraceptive utilization [13].

Although there are many reproductive health improvements, due to poor contraceptive utilization induced termination of pregnancy continues, causing about 13% of abortions in Ethiopia [14]. In Ethiopia, emergency contraceptives are distributed free of charge through health institutions and pharmaceutical markets with the support of non-governmental organizations but its utilization is poor.

Since termination of pregnancy is a sensitive reproductive health issue this study used a mixed quantitative and qualitative approach to explore women’s perspectives and reasons why women can prevent unwanted pregnancy by using contraceptives. Objectives of the study were (1) to determine women’s experiences of utilizing emergency contraceptives to prevent unwanted pregnancy; (2) to identify/explore the factors influencing emergency contraceptives utilization.

## Materials and methods

### Study setting and study period

An institution-based cross-sectional study triangulated with phenomenological approached in-depth interviews was carried out among pregnant women seeking termination of pregnancy from March 15 to May 15, 2019, in Northwest Ethiopia.

In the study area (Debre Marko’s town), three health centers, two non-governmental organizations (NGO) clinics, and one governmental specialized hospital all performed legal termination of pregnancy. Women seeking termination of pregnancy in those institutions were the study population; women who terminated for obstetrical reasons like fetal anomalies were excluded.

### Definitions

**Emergency contraceptives utilization:** use of an emergency contraceptive method within the last six months before the index pregnancy.

**Termination of pregnancy:** refers to deliberate intervention to terminate pregnancy.

**Good knowledge:** women who had mean scores ≥72% of contraceptive knowledge questions, were considered as having good knowledge.

**Positive attitudes:** women who had mean scores ≥72% of contraceptive attitude questions, were considered as having positive attitudes.

**Adverse obstetrical history of emergency contraceptives:** A woman who experienced failure of emergency contraceptive methods and/or got bad side effects in the last one year before index pregnancy.

**Accessibility:** if contraceptive methods were available within 2hr (30 km) on foot, family planning service provision was considered accessible [15].

### Sample size determination and sampling procedures

Sample size was calculated by using EPI-info with the assumptions of prevalence: 50%, margin of error: 5%, and 95% CI. By adding a 10% non-response rate, final sample size was 422. Based on the previous two months’ performance of health facilities that provide legal termination of pregnancy, proportional allocations were made. Systematic random sampling was implemented to select study participants. The interval of K was calculated by dividing each health institution’s two months client flow by required sample size allocated for each institution. Purposively 13 individuals were selected based on individuals’ age, marital status, and frequency of pregnancy termination for in-depth interviews.

### Data collection instruments and procedures

Interviewer-administered questionnaires were used for data collection. Questions were initially prepared in English and translated to the local language Amharic, and back translated to verify accuracy. Questionnaires contained socio-demographic and reproductive characteristics; and knowledge and attitude questions. Participant answers to questions were entered by nurses who were not directly involved in abortion care in each facility.

An in-depth interview was carried out by the principal investigator with one assistant from each health facility using an in-depth interview guide. Data quality was assured by doing pre-testing and training for data collectors and supervisors. Training was focused on data confidentiality, participants’ rights, informed consent, study objective, interview techniques and filling questionnaires.

### Data analysis

Knowledge questions were categorized and coded as good = 1 and poor = 0, whereas attitude questions were coded as 1 = positive and 0 = negative. Cronbach alpha was checked to assure internal consistency of attitudes measuring questions. Data were entered using EPI info and analyzed using SPSS version 23.

Descriptive statistics were used to describe data. Bivariate and multivariable logistic regression was performed to determine associations with outcome variables. P-value < 0.05 in multivariable logistic regression was considered statistically significant. Multicollinearity coefficients were assessed to check correlations with the independent variables. Model of fitness was checked by using Hosmer Lemshow test. For the qualitative study, data were transcribed, translated, coded, and thematically analyzed. We conducted descriptive thematic analysis based on the research questions and topic guides. Data were coded in Microsoft Excel.

#### Ethical considerations

Institutional Review Board (IRB) of Bahir Dar University had given ethical clearance to conduct this study (02012/18-09). A formal letter from the school of public health was given to the selected health institutions. Moreover, informed consent was obtained from each respondent and they were aware of their right to withdraw from the study at any time. Written informed consent was obtained from a parent or guardian for 16–18 years old participants. Participants were interviewed in a private room and assured that all information would be confidential.

## Results

A total of 422 were approached, and 397 (94.1%) participated. The majority were urban residents (314; 79.1%) and Orthodox religious followers (349; 87.9%). Mean age was 23.23 (SD ± 4.6) years with a predominant age group of 20–24 years (191; 48.1%). One-third of the women (135; 34.0%) completed secondary education and almost two-thirds (257; 64.7%) were not married (Table 1).

**Table 1:** Socio-demographic and sexual and reproductive characteristics of 397 women seeking termination of pregnancy, Northwest Ethiopia, 2019.

Variables		N	Percentage
Age of participants	15–19	80	20.2%
	20–24	191	48.1%
	25–29	67	16.9%
	> = 30	59	14.9%
Residence	Urban	314	79.1%
	Rural	83	20.9%
Religion	Orthodox	349	87.9%
	Muslim	25	6.3%
	Protestant	18	4.5%
	Others	5	1.3%
Marital status	Married	105	26.4%
	Unmarried	257	64.7%
	Divorced/Widowed	35	8.8%
Educational status	No formal education	56	14.1%
	Primary	100	25.2%
	Secondary	135	34.0%
	Tertiary & above	106	26.7%
Number of living children	0	302	76.1%
	≥1	95	23.9%
Age of first sexual debut	≤14	29	7.3%
	15–19	219	55.2%
	≥20	149	37.5%
Gestational age of current pregnancy	1st trimester	376	94.7%
	2nd trimester	19	4.8%
	3rd trimester	2	0.5%
Sexual partner discussion about ECs	No	268	67.5%
	Yes	129	32.5%
Adverse obstetrics history during EC utilization	No	314	79.1%
	Yes	83	20.9%
History of abortion	No	342	86.1%
	Yes	55	13.9%
Accessibility of EC methods	No	19	4.8%
	Yes	378	95.2%
Knowledge	poor knowledge	170	42.8%
	good knowledge	227	57.4%
Attitude	negative attitude	220	55.4%
	positive attitude	177	44.6%

Majority of the women’s (376; 94.7%) index pregnancy was in the first trimester. Most women (314; 79.1%) had no previous adverse obstetrics history when they used emergency contraceptives. Mean age of sexual debut was 18.3 (SD ± 2.6) years. Access to emergency contraceptives was almost complete (378; 95.2%) (Table 1).

More than half of the women (227; 57.2%) had good knowledge about emergency contraceptives and (117; 44.6%) women had positive attitudes towards emergency contraceptives (Table 1). Women’s awareness of emergency contraceptives was stated by a 23 year’s old unmarried woman: *“It is taken within 72 hours after unprotected sexual intercourse”*.

Almost one-fifth (78; 19.2%) of the participants used emergency contraceptives to prevent their index pregnancy (Table 2). Among the 78 women who used emergency contraceptive pills, (36; 46.2%) became pregnant as stated by a 23 year’s old unmarried woman: *“pregnancy occurred even when I used correctly emergency contraceptive pills”*.

**Table 2:** Use of emergency contraceptives to prevent the index pregnancy among 397 women seeking termination of pregnancy, Northwest Ethiopia, 2019.

		N	Percentage
Emergency contraceptive utilization to prevent the index pregnancy	No	319	80.4
	Yes	78	19.6
Post-abortion contraceptive utilization	No	28	7.1
	Yes	369	92.9

### Factors associated with emergency contraceptives’ utilization

Odds of emergency contraceptives’ utilization among women living in urban areas was 2.53 (95% CI: 1.01–6.31) as compared to those in rural areas. Women who had secondary education had higher odds of emergency contraceptive utilization (aOR 3.28; 95% CI: 1.59–6.79 as compared to those with tertiary and above education. Women who had no living children had higher odds of emergency contraceptives utilization (aOR 4.52; 95% CI: 1.40–14.57) compared to those who had.

Odds of emergency contraceptive utilization among women who had not discussed about contraceptives with their sexual partners was lower (aOR 0.49; 95% CI: 0.27–0.91) than among women without discussion. Women who had no history of abortion had lower odds of emergency contraceptive utilization (aOR 0.45; 95% CI: 0.24–0.97) than those with such history (Table 3).

**Table 3:** Factors associated with emergency contraceptive utilization among 397 women seeking termination of pregnancy, Northwest Ethiopia, 2019.

Variables		EC utilization			AOR (95% CI)
		Yes	No		
Age	15–19	10	70		0.52(0.09–2.71)
	20–24	52	139		1.99 (0.47–8.49)
	25–29	13	54		2.62 (0.63–10.96)
	> = 30	3	56		1
Occupation	Merchant	5	54		1
	Student	43	116		6.26(1.72–22.72)
	house wife	1	42		0.37 (0.04–3.79)
	private employ	9	30		3.87(0.98–15.21)
	Other	20	77		5.56(1.66–18.65)
Residence	Urban	71	243		2.53 (1.01–6.31)
	Rural	7	76		1
Marital Status	Unmarried	63	194		0.79(0.19–3.16)
	Married	10	95		0.31(0.07–1.45)
	divorced/widowed	5	30		1
Educational status	no formal	3	50		1.22(0.27–5.49)
	primary	15	87		2.31(0.89–5.99)
	secondary	42	104		3.28(1.59–6.79)
	tertiary and above	18	78		1
Knowledge about contraceptives	poor knowledge	20		150	0.61(0.30–1.25)
	good knowledge	58		169	1
Attitude towards contraceptives	negative attitude	49		171	1.19(0.65–2.21)
	positive attitude	29		148	1
Number of living children	0	73		229	4.52(1.40–14.57)
	> = 1	5		90	1
Contraceptive accessibility	No	42		217	0.18(0.02–1.55)
	Yes	36		102	1
Discussion with partner	No	1		18	0.49(0.27-.91)
	Yes	77		301	1
History of abortion	No	62		280	0.45(0.20–0.97)
	Yes	16		39	1

**Fig 1:**
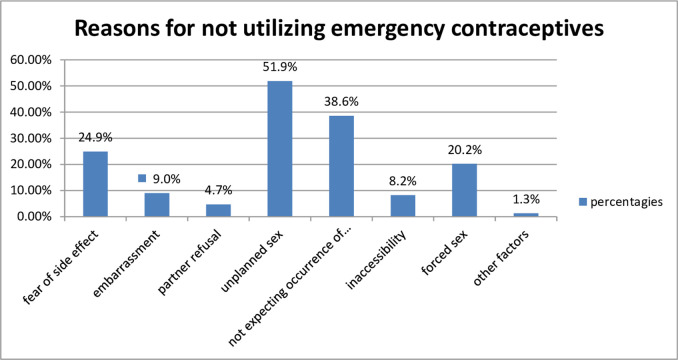
Reasons of not utilizing emergency contraceptives of women seeking termination of pregnancy, Northwest Ethiopia, 2019(n = 319).

Participants’ main reason for not using ECs to prevent their index pregnancy was unplanned sex. More than half (121, 51.9%) of the participants explained that their pregnancy was due to unplanned sex (Fig 1) which is supported by the in-depth interview result *“I did not use any contraceptive since I have no boyfriend*, *I didn’t expect to have sex but incidentally I have sex with my classmate which makes me frustrated and disturbed on the situation*, *and I forgot to uptake even ECs”* (18 years unmarried women).

More than one-third of the women (90; 38.6%) did not expect their index pregnancy to occur since they were lactating at the time of their conception (Fig 1). This was supported by the in-depth interview of one of the women: *“When I was an adult*, *my family said that breastfeeding prevents pregnancy for two years after childbirth even when unprotected sex occurred in between…since I was a lactating mother of a one year child*, *I did not expect pregnancy”* (25 years old woman).

*“The pregnancy has occurred even I used a 28 tablets oral contraceptive regularly” said a* 23 year’s old unmarried woman. The women answered for the question, what did you think behind the occurrence of pregnancy even you used contraceptives? She said that “since I used emergency contraceptive pills correctly I doubt its effectiveness.”

Effectiveness of emergency contraceptives also compromised by women’s poor understanding on instructions how to take EC pills: “….*my husband comes on Friday*, *and we have sex for the following four days but I have taken EC on the 5*^*th*^ *day”* (A 25 years old married woman).

## Discussion

Unintended pregnancy is the main reproductive health problem especially for youth in low income countries, and utilization of emergency contraceptives is an effective intervention to prevent such pregnancies when taken within 72 hours after unprotected sexual intercourse. Our major finding is the relatively low emergency contraceptive utilization (19.6%) with a high failure frequency of emergency contraception (46.2%). Good knowledge regarding emergency contraceptives was observed in 57.2% of the women, although lower than in Ghana (69%) and South Africa (70%), perhaps due to socio-demographic differences between the study areas [8, 16].

On the contrary women’s knowledge of emergency contraceptives was higher than found in South Africa (15%), Delhi (34%) and India (5.5%) [8, 17, 18]. Possible explanation is a collaborative effort of health professionals to create better contraceptive awareness in Ethiopia [19]. Our study also targeted women seeking medical termination of pregnancy who also were aware and committed to getting reproductive health care.

Women’s knowledge was also higher than found in Jimma (10.1%), Eastern Tigray (40.4%) and Dire Dawa (34.1%). This may be due to improvement in women’s awareness since these earlier studies [20–22].

Emergency contraceptive utilization among women seeking induced termination of pregnancy is lower than utilization level of regular contraceptive methods. Misconceptions regarding the action of EC may limit its use [23, 24]. Level of emergency contraceptive utilization in this study is much lower than the study done in Delhi (79%), South Africa (40%), Dessie (51%) and Shanghai (51.2%) [8, 18, 25, 26]. The difference in the study population and access to contraceptive services might be the reason for low emergency contraceptive prevalence in the current study as compared with the above listed reports.

Our findings show higher EC utilization percentage than in Eastern Tigray(12.2%), Dire Dawa (9.7%) and Jimma (5.4%) [8, 22]. Perhaps knowledge differences could explain this since 57% of our study participants had good knowledge about emergency contraceptives. Another possible reason is participants’ residence; urban dwellers women have 2.53 times higher emergency contraceptives utilization than rural residents [20].

Women who had no living children were more likely to utilize emergency contraceptives as compared with their counterparts, similar to Shanghai, where primiparous women had better emergency contraceptive utilization than multiparas. Perhaps women without children could be unmarried and had higher motivation to prevent unwanted pregnancy [26, 27]. Women living in urban areas had increased odds of emergency contraceptives utilization as compared to those in rural areas which is supported by the study in Cape Town [28]. Higher emergency contraceptives utilization was observed among women who discussed contraception with their partners, consistent with other studies in Bahir Dar and North Showa [29, 30].

Social desirability bias is one of the limitations of this study since women may report more acceptable responses. Recall bias might have also occurred since women may forget the exact time of the current conception. Since the study design is cross sectional study, it didn’t establish a cause-and-effect relationship between the independent and outcome variables.

## Conclusions

Even though emergency contraceptives can prevent unwanted pregnancy and reduce induced terminations of pregnancy, limitations exist to practice preventive measures of unwanted pregnancy including emergency contraception. In our study, there was a relatively low utilization with a high failure frequency of emergency contraception.

Improving the educational status of the women, good parental discussion about emergency contraceptives, history of previous abortion and urban residence are the protective factors on women’s emergency contraceptive utilization. On the contrary presence of live children negatively affects women’s emergency contraceptive utilization.

Reproductive health programs further encourage women discussion with their partners about emergency contraceptives to decrease occurrence of unwanted pregnancy and termination of pregnancy. The family welfare program should create sustainable awareness about emergency contraceptives in addition to regular contraceptives, this shall certainly empower women to control their fertility and hence in essence their lives.

Further studies will be needed to explore reasons for low uptake of contraceptives despite women having knowledge about emergency contraceptives. This study collects the data only from pregnant women presenting for termination of pregnancy; so further studies also needed to investigate the perspectives of sexual partners, nurses and doctors on emergency contraceptive utilization.

## Supporting information

S1 Questionnaire (DOCX)

## Abbreviations

AOR: Adjusted Odds Ratio
CI: Confidence Interval
COR: Crude Odds Ratio
EC: Emergency Contraceptives
HIV: Human Immune Virus
IUCD: Intra-Uterine Contraceptive Device
NGO: Non-Governmental Organization
SD: Standard Deviation
SPSS: Statistical Package for the Social Sciences
STD: Sexually Transmitted Disease
WHO: World Health Organization
