# Supplementary material for: Practice and determinants of emergency contraceptive utilization among women seeking termination of pregnancy in Northwest Ethiopia—A mixed quantitative and qualitative study
Source: PLoS One. 2022 Feb 11;17(2):e0263776. doi: 10.1371/journal.pone.0263776 (PMC8836300; doi:10.1371/journal.pone.0263776)
Supplement: S1 Questionnaire — (DOCX) [file pone.0263776.s001.docx]

**Questionnaires for women who are seeking termination of pregnancy
Code No-------------------------------Date of interview------------------------------------
Interviewer name --------------------Sig --------Name of supervisor----------------Sig----**

**Section A: Socio-demographic status of the respondent**

| NO | Questions | Answer | Skip |
| --- | --- | --- | --- |
| 101 | Age in years | -------------(years) |  |
| 102 | Residence | 1. Urban 2. Rural |  |
| 103 | Marital status | 1. Unmarried 2. Married/in-union 3. Divorced 4. Widowed/separated |  |
| 104 | Religion | 1. Orthodox 2. Muslim 3. Protestant 4. Catholic 5. Others |  |
| 105 | Educational status | 1. No formal education 2. Primary education 3. Secondary education 4. Tertiary (Univ & College) |  |

**Section B: obstetrical and Sexual history of the respondent**

| NO | Questions | Answer | Skip |
| --- | --- | --- | --- |
| 201 | Age at first sex | ---------------- |  |
| 202 | Number of live children | ------------- |  |
| 203 | Gestational age of the current pregnancy | ------------------ |  |
| 204 | Have you history of abortion ever? | 1. Yes 2. No | If No skip Q-205 |
| 205 | If yes how many | 1. One 2. Two 3. Three and above |  |
| 206 | Do you have discussion with your sexual partner about EC? | 1. Yes 2. No |  |

**Section C: Knowledge about contraceptives of the respondent**

| NO | Questions | Answer | Skip |
| --- | --- | --- | --- |
| 301 | Do you know types of contraceptives methods? | 1. Yes 2. No | If No, skip Q-302 and Q-303 |
| 302 | If yes for Q-301, which methods do you know?(if she knows at least one method, consider the question as correct)  (multiple choice is possible) | 1. Natural 2. Condom 3. Oral contraceptives 4. Injectable contraceptive 5. Implanon 6. IUCD 7. Others(specify)______ |  |
| 303 | What is the source of your information(if she knows at least one method, consider the question as corr3ect)  (multiple choice is possible) | 1. Health personnel 2. Radio/TV 3. Friends 4. Local news/newspaper/ magazines 5. Others(specify)________ |  |
| 304 | Do you know the importance of EC? | 1. Yes 2. No | If No, skip Q-305 |
| 305 | If yes, what is the importance?(if she knows at least one importance, consider the question as correct) (multiple choice is possible) | 1. Prevent unwanted pregnancy 2. Limiting number of children 3. Spacing of children 4. Others(specify)________ |  |
| 306 | Do you know the place where you can get family planning service? | 1. Yes 2. No | If No, skip Q-307 |
| 307 | If yes, where family planning services are being offered?  (if she knows at least one method, consider the question as correct) (multiple choice is possible) | 1. Public health institutions 2. Private health institutions 3. Social marketing 4. Others(specify)_______ |  |
| 308 | Do you know about emergency contraceptive | 1. Yes 2. No | If No, skip Q-309 |
| 309 | If Yes, when it will be taken after sex | ------------- |  |

**Section D: Attitude about contraceptives of the respondent**

| NO | Questions | Answer | Skip |
| --- | --- | --- | --- |
| 401 | Do you believe EC utilization not benefits to improve maternal health? | 1. Strongly agree 2. Agree 3. Neutral 4. Not agree 5. Strongly not agree |  |
| 402 | Do you believe family planning service room is convenient? | 1. Strongly agree 2. Agree 3. Neutral 4. Not agree 5. Strongly not agree |  |
| 403 | Do you believe family planning service working hour not is convenient for you? | 1. Strongly agree 2. Agree 3. Neutral 4. Not agree 5. Strongly not agree |  |
| 404 | Do you believe family planning service working day is convenient for you? | 1. Strongly agree 2. Agree 3. Neutral 4. Not agree 5. Strongly not agree |  |
| 405 | Do you think family planning service providers’ are not judgmental? | 1. Strongly agree 2. Agree 3. Neutral 4. Not agree 5. Strongly not agree |  |
| 406 | Do you believe EC utilization reduce governmental cost for induced abortion? | 1. Strongly agree 2. Agree 3. Neutral 4. Not agree 5. Strongly not agree |  |
| 407 | Do you believe on misconception about contraceptive methods rumored by the community? | 1. Strongly agree 2. Agree 3. Neutral 4. Not agree 5. Strongly not agree |  |
| 408 | Do you believe the effective EC utilization reduce rate abortion | - - - 1. Strongly agree       2. Agree       3. Neutral       4. Not agree       5. Strongly not agree |  |
| 409 | Do you consider abortion as contraceptive method? | 1. Strongly agree 2. Agree 3. Neutral 4. Not agree 5. Strongly not agree |  |

**Section E: Organizational status of the family planning service**

| No | Questions | Answer | Skip |
| --- | --- | --- | --- |
| 501 | Do you have any contraceptive delivery center within 2 hours journey by foot (30Km) from your home? | 1. Yes 2. No |  |

**Section F: Contraceptives utilization history of the respondent**

| No | Questions | Answer | Skip |
| --- | --- | --- | --- |
| 601 | Since the last 6 months, have you ever used any contraceptive method avoid the current pregnancy? | 1. Yes 2. No | If no skip Q-602; If yes skip Q-603 |
| 602 | If yes, which type of contraceptive you used recently?  (multiple choice is possible) | 1. COC 2. POP 3. Injectable 4. Implanon 5. IUCD 6. Condom 7. Others(specify)______ |  |
| 603 | If no, what are the factors for not utilizing any contraceptive methods? (multiple choice is possible) | 1. Fear of side effects 2. Embarrassment to take contraceptives 3. Sexual partner refusal 4. Unplanned sex 5. Not expecting occurence of pregnancy 6. Not accessible at a time 7. Forced sex 8. Others(specify)__________ |  |
| 604 | Did you have any bad history of contraceptive utilization? | 1. Yes 2. No |  |
| 605 | Have you ever used anything any short term contraceptive methods avoid the current pregnancy during sex on which you suspected to conceive? | 1. Yes 2. No | If no skip Q-606 |
| 606 | If yes, which method or methods are you using?  (multiple choice is possible) | 1. Withdrawal 2. condom 3. others(specify)_______ |  |
| 607 | Did you use emergency contraceptive after sex to prevent the current pregnancy? | 1. Yes 2. No | If yes skip Q-608 |
| 608 | If No, what is the reasons not utilizing emergency contraceptive to prevent the current pregnancy?  (multiple choice is possible) | - 1. Have drunk when I conceive the current pregnancy   2. Denial of pregnancy   3. Not accessible at a time   4. Others(specify)_______ |  |
| 609 | Do you have desire to use contraceptive methods after the procedure? | 1. Yes 2. No |  |

**Annexes2: Qualitative Data- interview guide**

Hello. My name is ___________________ and I am studying MPH/RH in BDU, I would like to talk to you about your experience of using EC methods. I am interested in your opinions because we are trying to find out how you fell about getting FP services at this health facility. I would like to ask you a few questions that should not take more than 30 minutes. I will not write down your name and everything you tell me will be kept strictly confidential.

Site: health institutions abortion room Date: -------------------------

Interviewer: principal investigator Age: ------------------- Time: -------------------- Educational level--------------

Marital Status: ----------------- Occupation----------------------

History of previous abortion---------- Code: ----------------

1. Please tell me what you know about EC?
2. What do you say about the importance of EC to reduce rate of termination of pregnancy?
3. Where do you get EC?
4. Are the working days and working hours of the health facility convenient for you?
5. What do you think about the increment of induced abortion despite accessible EC methods?
6. Why you are not utilizing EC methods prior to having sex to prevent the current unwanted pregnancy?
7. Why you are not tried to use emergency contraceptives after sex to prevent the current pregnancy?
